# Clindamycin and intravenous immunoglobulins in pediatric invasive group A streptococcal disease: what is the evidence?

**DOI:** 10.1007/s00431-026-06797-7

**Published:** 2026-02-20

**Authors:** Evelien B. van Kempen, Annika Malmgren, Kristina Elfving, Jérémie F. Cohen, Dorine M. Borensztajn

**Affiliations:** 1 Department of Paediatrics, Erasmus Medical Center Sophia, Dr. Molewaterplein 40, 3015 GD Rotterdam, the Netherlands; 2 Department of Paediatrics, Juliana Children’s Hospital, Haga Hospital, the Hague, the Netherlands; 3 Department of Paediatrics, Queen Silvia Children’s Hospital, Gothenburg, Sweden; 4 Department of Pediatrics, Institution for Clinical Sciences, Sahlgrenska Academy, University of Gothenburg, Gothenburg, Sweden; 5 Department of General Pediatrics and Pediatric Infectious Diseases, Hôpital Necker-Enfants Malades, AP-HP, Université Paris Cité, Paris, France; 6 Department of Paediatrics, Northwest Clinics, Alkmaar and Den Helder, the Netherlands

**Keywords:** Group A streptococcal disease, *Streptococcus pyogenes*, Infectious diseases, Adjunctive treatment, Clindamycin, IVIG

## Abstract

Invasive group A streptococcal disease (iGAS) can present with severe manifestations, including sepsis, necrotizing fasciitis, and streptococcal toxic shock syndrome. Although β-lactam antibiotics are considered standard therapy, the role of adjunctive treatments, particularly clindamycin and intravenous immunoglobulin (IVIG), in children with these severe manifestations remains debated. Clindamycin is effective irrespective of pathogen load and is suggested to inhibit toxin production, while IVIG is hypothesized to modulate the immune response through multiple mechanisms, including neutralization of superantigens. The rarity of iGAS in children and the relatively low pediatric mortality rate (2–10%) complicate gathering evidence, with most data derived from small, low-quality studies. Evidence is often extrapolated from adult studies, despite substantial differences in mortality rates (30–80%). In this narrative review, we discuss the available evidence on the use of adjunctive therapies in severe pediatric iGAS and its possibility in guiding clinical management. *Conclusion:* Current evidence does not allow definitive conclusions regarding the benefit of clindamycin or IVIG in severe pediatric iGAS. Treatment decisions therefore largely rely on expert consensus and individualized clinical judgment. Ongoing multicenter studies, such as PEGASUS, aim to clarify the role of these adjunctive therapies and guide evidence-based practice.

**Table:** 

**What is Known:** • *β-Lactams are first-line treatment for pediatric invasive group A streptococcal disease (iGAS)*. • *Clindamycin and IVIG have been suggested as adjuncts in severe iGAS, but recommendations differ, and many countries have no formal guidance*.	
**What is New:** • *This review shows that evidence supporting clindamycin and IVIG in children with iGAS remains limited and is largely extrapolated from adult studies. It highlights variability in clinical practice, safety and cost concerns, and the need for larger-scale pediatric studies*.

**What is Known:**

• *β-Lactams are first-line treatment for pediatric invasive group A streptococcal disease (iGAS)*.

• *Clindamycin and IVIG have been suggested as adjuncts in severe iGAS, but recommendations differ, and many countries have no formal guidance*.

**What is New:**

• *This review shows that evidence supporting clindamycin and IVIG in children with iGAS remains limited and is largely extrapolated from adult studies. It highlights variability in clinical practice, safety and cost concerns, and the need for larger-scale pediatric studies*.

## Introduction

Group A *Streptococcus* (GAS) is a Gram-positive, β-hemolytic, facultative anaerobic coccus that spreads via droplets, (in)direct contact, and possibly airborne transmission. About 10% of school-aged children are asymptomatic pharyngeal GAS carriers, compared to 1–5% of adults.

GAS causes a broad spectrum of infections, most commonly non-invasive diseases like pharyngitis and impetigo. Sometimes, GAS causes invasive infections (iGAS), defined as the presence of bacteria in a normally sterile site (e.g., pneumonia, osteomyelitis, bacteremia, and meningitis) or with severe manifestations such as sepsis, necrotizing fasciitis (NF), or streptococcal toxic shock syndrome (STSS). STSS, mediated by superantigens that trigger a massive T-cell response, is defined as iGAS disease with hypotension and organ failure. Why some children develop iGAS disease while others only experience non-invasive disease remains poorly understood. It is likely an interplay involving host factors (e.g., genetic susceptibility and absence of prior immunity), pathogen characteristics (e.g., *emm* type and other virulence factors), inoculum size, and viral co-infections with varicella-zoster or influenza.

Recently, there has been renewed interest in identifying the optimal treatment strategy for iGAS disease in children. This is due to the post-COVID-19 pandemic surge in iGAS, with a disproportionate burden in children [1, 2]. While β-lactams are the standard treatment, the role of clindamycin and intravenous immunoglobulins (IVIG) as adjunctive treatments remains unclear, even though severe iGAS cases are associated with substantial morbidity and mortality [3, 4]. Some guidelines suggest adjunctive therapy for all children with iGAS disease, whereas others limit it to cases presenting with NF or shock [3]. For many countries, no formal recommendations exist [3]. Here, we provide a narrative review of the evidence regarding the use of clindamycin and IVIG in children with iGAS disease.

## Methods

A structured literature search on PubMed was conducted in October 2025 (Box 1). Additional studies were identified through snowballing from reference lists. We reviewed studies reporting outcomes of clindamycin and/or IVIG in children or adults with iGAS.

**Table:** **Box 1**. Search strategy

("Streptococcus pyogenes"[Mesh] OR streptococcus pyogenes[tiab] OR "group A streptococ*"[tiab] OR streptococcal[tiab] OR ("Streptococcal Infections"[Mesh] AND (group A[tiab] OR pyogenes[tiab]))) AND (invasive[tiab] OR bacteremi*[tiab] OR sepsis[tiab] OR "toxic shock"[tiab] OR “STSS”[tiab] OR "necrotizing fasciitis"[tiab] OR “necrotising soft tissue infection”[tiab]) AND ("Clindamycin"[Mesh] OR clindamycin[tiab] OR "Immunoglobulins, Intravenous"[Mesh] OR "intravenous immunoglobulin*"[tiab] OR IVIG[tiab] OR “immunoglobulin G”[tiab])	

## Results

An overview of the characteristics and results of the included studies can be found in Table 1.

**Table 1:** Overview of the characteristics and results of included studies

Author; country	Study type	Population	Sample size	Year of data collection	Year of publication	Comparison	Results	Limitation
**Clindamycin**								
Mulla [7]; USA	Retrospective population-based study	Children and adults with iGAS Median age 52 years (range: 0–103)	Total: 257	1996–2000	2003	β-Lactam plus clindamycin vs only β-lactam	Mortality: overall 18%, in group < 55 years 12.4% Reduced mortality with clindamycin in patients with NF (OR 0.11, 95% CI 0.01–0,89), but not iGAS without NF (OR 1.01, 95% CI 0.31–3.33)	Majority of population likely adults (high median age)
Carapetis [8]; Australia	Nationwide prospective surveillance study	Children and adults with iGAS Median age clindamycin group 56.2 years (range: 3.8–88.1) and no-clindamycin group 70.4 years (range: 2.6–96.2)	Total: 333 *N* = 84 with severe iGAS (i.e., STSS, NF, septic shock, or cellulitis with hypotension)	2002–2004	2014	β-Lactam plus clindamycin vs only β-lactam	*Clindamycin in 53 (63%) cases* Clindamycin-treated patients had more severe disease (i.e., ICU admission, presence of STSS and length of hospital stay) compared to clindamycin-untreated patients, but lower mortality (15% vs 39%) (*P* = 0.014); OR 0.28 (95% CI 0.10–0.80)) Adjusted point estimate of the OR for mortality (adjusted for age and presence of STSS): lower in clindamycin-treated patients (aOR 0.31, 95% CI 0.09–1.12)	Underpowered No statistical significance Majority of population likely adults (high median age)
Babiker et al. [9]; USA	Retrospective multicenter cohort study	Adults with iGAS (and invasive non-group A/B β-haemolytic streptococcal infections)	Total: 1956 *N* = 1079 with iGAS	2000–2015	2021	β-Lactam plus clindamycin vs only β-lactam	*Clindamycin in 343/1079 (31.7%) patients* In-hospital mortality, crude: 6.5% vs 11.0% (*P* = 0.04) In-hospital mortality, adjusted: aOR 0.44 (95% CI 0.23–0.81) In-hospital mortality, subgroup without vasopressor-dependent shock or necrotizing fasciitis: 2.6% vs 6.1% (*P* = 0.04; aOR 0.40 (95% CI 0.15–0.91))	Adults only
Linner et al. [10]; Sweden	Nationwide surveillance study	Adults with STSS	67	2002–2004	2014	β-Lactam plus clindamycin vs only β-lactam	*Clindamycin in 52/67 (78%) of patients* Survival: OR 8.6 (95% CI 1.8–40.4, *P* = 0.007) with clindamycin	Adults only
Zimbelman [11]; USA	Retrospective study	Children with iGAS	Total: 56 *N* = 19 with “deep infection” (= necrotizing fasciitis, bacteraemia, arthritis, osteomyelitis) *N* = 37 with “superificial disease”	1983–1997	1999	β-Lactam plus clindamycin vs only β-lactam	Favorable outcome in 83% vs. 14% (*P* = 0.006) with “deep infection” if clindamycin-treated	Small sample size Better outcome towards end of data collection period (improved medical care in general?) Inclusion of NF, where clindamycin seems to have a good effect
**IVIG**								
Senda et al. [18]; Japan	Nationwide observational study	Adults with iGAS Median age 65 years	481	2018–2021	2023	β-Lactam plus clindamycin plus IVIG vs β-lactam plus clindamycin	Overall mortality: 31.0% In-hospital mortality: no difference (aOR 0.99, 95% CI 0.93–1.04, *P* = 0.92) Similar results after propensity score matching (OR: 1.00, 95% CI 0.62–1.61, *P* > 0.99)	Adults only
Linnér et al. [10]; Sweden	Nationwide surveillance study	Adults with STSS Median age IVIG group 60 years (range: 31–87) and no-IVIG group 65 years (range: 32–92)	67	2002–2004	2014	β-Lactam* (+/− clindamycin) plus IVIG vs β-lactam* (+/− clindamycin) *one patient received no β-lactam (group unspecified)	*IVIG in 23/67 (34%) of patients. 21/23 (91%) received clindamycin* 28-day survival: 87% vs 50% (*P* < 0.01; OR 6.7 (95% CI 1.7–25.7)) Factors influencing survival in STSS: Simplified Acute Physiology Score II (OR 1.1; *P* = 0.007), clindamycin (OR 8.6; *P* = 0.007), and IVIG (OR 5.6; *P* = 0.030)	Adults only
Madsen [20]; Scandinavia (1 hospital in Denmark, 3 hospitals in Sweden, 1 hospital in Norway)	RCT	Adult patients with necrotising soft tissue infection at, or planned to be admitted, to ICU	100	2014–2016	2017	Standard therapy* plus IVIG vs standard therapy* plus placebo *Standard therapy = *meropenem, clindamycin* *and ciprofloxacin*	Mortality, day 28: 12% in IVIG compared to 12% in placebo (6/50 each; RR 1.0, 95% CI 0.35–2.89; *P* > 0.99) Physical component summary score of Short Form Health Survey (SF-36) at 6 months: 36 (IQR 0–43) in IVIG vs 31 (IQR 0–47) in placebo; mean adjusted difference 1 (95% CI − 7 to 10), *P* = 0.81	Pathogen not specified
Darenberg et al. [21]; Europe	Placebo-controlled RCT	Adults with STSS	21	1999–2001	2003	β-Lactam plus clindamycin plus IVIG vs β-lactam plus clindamycin plus placebo	Mortality at 28 days: 10% (1/10) vs 36% (4/11) Decreased SOFA score at days 2 (*P* = 0.02) and 3 (*P* = 0.04) Increased plasma neutralizing activity against superantigens (*P* = 0.03)	Adults only Underpowered
Carapetis [8]; Australia	Nationwide prospective surveillance study	Children and adults with iGAS Median age not mentioned	Total: 333 *N* = 84 with severe iGAS (i.e., STSS, NF, septic shock, or cellulitis with hypotension)	2002–2004	2014	β-Lactam plus clindamycin plus IVIG vs β-lactam plus clindamycin	*Clindamycin in 53/84 (63%) cases* *IVIG in 14/84 (17%) case*s, *all of whom also received clindamycin. In the IVIG-group, all had STSS and/or NF* Mortality: 7% vs 18% (*P* = 0.24) Adjusted point estimate of the OR for mortality (adjusted for age and presence of STSS): aOR 0.12 (95% CI 0.01–1.29) vs aOR 0.39 (95% CI 0.10–1.46)	Underpowered No statistical significance Majority of population likely adults (high median age)
Parks et al. [22]	Systematic review and meta-analysis	Adults and children with STSS	160 Includes [5, 7, 11, 13] and Kaul et al. (1999)	1980–2017	2018	IVIG vs no-IVIG	5 studies individually: administration of IVIG in the clindamycin-treated subgroup associated with lower mortality rates, yet no statistical significance Pooled analysis: IVIG associated with reduction in mortality rate (from 33.7% to 15.7%; RR 0.46, 95% CI 0.26–.83; *P* = 0.01), consistent across nonrandomized studies (RR 0.47; 95% CI 0.25–0.86) and effect size estimate of the RCT (RR 0.42; 95% CI 0.05–3.28)	Small sample size Effect size estimate has large CI Baseline characteristics probably differing Majority of population adults
Adalat [23]; UK	Survey-based multicenter retrospective study	Children with streptococcal and staphylococcal TSS	Total: 49 *n* = 29 with streptococcal TSS *N* = 20 with staphylococcal TSS	2014	2014	N.A	Mortality: 8/29 (27.6%) *Clindamycin in 3/8 (38%) of patients who died and in 30/41 (73%) of survivors* *IVIG in 0/8 (0%) of patients who died, and in 10/41 (24%) of survivors*	Underpowered study with methodological limitations, limiting reliability and generalizability
Shah [16]; USA	Multicenter retrospective cohort study	Children with STSS	192	2003–2007	2009	Penicillin or vancomycin (+/− clindamycin) plus IVIG vs penicillin or vancomycin (+/− clindamycin)	*Clindamycin in 182/192 (94.8%)* *IVIG in 84/192 (44%). Use was stable over time, varied by hospital (29–60%), and given to 30% of non-ICU patients)* Mortality, overall: 6% (IVIG) vs 2.8% (no-IVIG) (*P* = 0.300). Propensity-matched mortality: 4.5% in both groups (*P* = 1.000) Length of stay: no significant difference Hospital costs: increased in IVIG group (median difference $6139)	Identification of STSS cases relied on discharge ICD codes and billing for IV penicillin, making it likely that some patients without STSS were included

### Clindamycin

In pediatric practice, clindamycin is used to treat anaerobic, streptococcal, and staphylococcal infections, including skin, soft tissue, bone, and joint infections. It is a lincosamide antibiotic that inhibits protein synthesis by binding to the 50S subunit of the bacterial ribosome, thereby preventing peptide chain elongation. This not only halts bacterial growth but also inhibits production of virulence factors and exotoxins, including superantigens. While β-lactams rely on active cell wall synthesis, which could be reduced in high-inoculum infections due to altered bacterial physiology and slowed growth—a phenomenon historically associated with the *Eagle effect—*clindamycin remains effective regardless of bacterial growth phase [5]. This makes it particularly valuable in infections with high bacterial load.

#### Clindamycin in mouse models, adults, and mixed-age populations

For NF, clindamycin significantly reduces skin lesions in mice [6]. This benefit likely transfers to humans, where it possibly also decreases mortality. Mulla et al. performed a retrospective analysis of 228 patients, mostly adults, with iGAS in the USA [7]. Of the 45 patients with NF, treatment with clindamycin was associated with significantly lower mortality (OR, 0.11). No benefit was observed in patients with iGAS without NF. In 2014, Carapetis et al. performed a population-based, prospective, active surveillance study of iGAS in Australia [8]. Data were collected between 2002 and 2004 and included 84 cases with severe iGAS disease. Clindamycin-treated patients had more severe disease but a lower mortality rate (15% vs 39%), though not significantly. It is likely that the number of children was low, as the median ages of the two groups were 56 and 70 years. The retrospective multicenter cohort study by Babiker et al., including 1000 adults with iGAS between 2000 and 2015, showed that clindamycin use was associated with significantly lower in-hospital mortality, even in patients without shock or NF (6.5% versus 11%) [9]. For STSS, one of the main publications is the prospective surveillance study by Linnér et al., including 67 adults. They noted a significant benefit on survival with the addition of clindamycin to penicillin (aOR, 8.6) [10].

#### Clindamycin in children

In 1999, Zimbelman et al. showed a favorable outcome in 83% of clindamycin-treated children with iGAS, compared to 14% if treatment consisted of β-lactams alone [11]. This was a small, retrospective study, with only 19 children with “deep infection,” including NF. Moreover, children admitted towards the end of the data collection (1983–1997) generally had a better outcome, likely due to improved medical care. The true effect of clindamycin is therefore difficult to isolate.

In 2021, Laho et al. published a review of the benefit of clindamycin in pediatric iGAS, based on the studies by Zimbelman and Carapetis [3]. They concluded that “despite the lack of scientific evidence, the drug is safe and inexpensive” and proposed its use to treat “all serious iGAS infections which need to be treated in a hospital.” This statement on safety contrasts with publications regarding an increase in *Clostridioides difficile*–associated diarrhea, with clindamycin being a high-risk drug. Furthermore, the increasing antibiotic resistance among GAS isolates, mostly due to *erm* genes, is concerning. Notably, this resistance is frequently cross-induced by macrolide exposure, as macrolides share an overlapping ribosomal target. Consequently, macrolide-resistant isolates may also exhibit clindamycin resistance, underscoring the importance of antimicrobial stewardship when prescribing these agents in children [12]. Importantly, clindamycin has been shown to decrease toxin production as well as skin lesion size in mice with NF, irrespective of the isolate susceptibility [6].

#### Other protein inhibitors

An ongoing discussion concerns the use of linezolid, an oxazolidinone that also inhibits protein synthesis via the 50S ribosome subunit, as an alternative to clindamycin. Resistance towards linezolid is lower for GAS, it crosses the blood–brain barrier, short-term use is generally safe in children, and it appears to be as effective as clindamycin in retrospective studies in adults [13–15].

In summary, the clinical effect of clindamycin in severe iGAS has been demonstrated in studies involving adults and mixed-age populations with a median age above 50 years. The only study focusing exclusively on children is small and from 1999.

### IVIG

In pediatric practice, IVIG is used to treat immunoglobulin deficiencies, but also to modulate the inflammatory process, for example in Kawasaki disease. IVIG acts by (a) enhancing bacterial clearance through opsonization and phagocytosis; (b) neutralization of exotoxins, including superantigens; (c) inhibition of T-cell proliferation and cytokine production; and (d) exertion of a broader immunomodulatory effect [10, 16, 17]. For iGAS, IVIG could help mitigate the massive “cytokine storm” that occurs when GAS superantigens are at play. When IVIG is administered, it is usually in addition to clindamycin, and limited data exists on the addition of IVIG without clindamycin.

#### IVIG in mouse models, adults, and mixed-age populations

Few studies have evaluated IVIG in patients with iGAS without STSS, and those that have been conducted did not show reduced in-hospital mortality [18]. Studies on IVIG in NF have concluded that penetration into necrotic tissue might be limited, and mouse models failed to demonstrate an effect on the course of the disease [19]. Linnér et al. observed a positive, albeit non-statistically significant, benefit of IVIG on survival in adult patients with NF [10]. These patients also had STSS, which makes interpretation difficult. A randomized controlled trial (RCT) published in 2018 on 100 adults with NF showed no difference in 30-day mortality nor serious adverse events between IVIG and placebo groups [20]. However, the causative pathogens were not specified. Overall, evidence supporting IVIG in iGAS other than STSS is not convincing. The important question that remains is whether to use IVIG in STSS.

Darenberg et al. designed a European multicenter, double-blind RCT to provide a definite answer on the efficacy and safety of IVIG in STSS [21]. Patients received either IVIG or placebo for 3 days, in addition to clindamycin and benzylpenicillin. Of 21 adults enrolled, 10 received IVIG, before early termination due to low enrollment. Patients who received IVIG demonstrated decreased sepsis-related organ failure assessment (SOFA) score at days 2 and 3 and increased plasma neutralizing activity against superantigens. The 28-day mortality rate was lower in the IVIG group (1/10) compared with placebo (4/11), yet not statistically significant, probably due to the small sample size. The question was therefore left unresolved. Some co-authors subsequently performed a prospective analysis in 2014 of 67 adults with STSS in Sweden [10]. This study showed significantly improved 28-day survival in the IVIG group (87% vs 50%).

Also in 2014, Carapetis et al. evaluated the effectiveness of IVIG in 84 cases of severe iGAS in Australia [8]. It is unclear how many of these were children. IVIG was used in addition to clindamycin and a β-lactam in 14 patients. Of these, 13 had STSS. This study suggested a lower, yet not statistically significant mortality rate among patients receiving IVIG in addition to a β-lactam.

#### IVIG in children

A 2018 systematic review and meta-analysis including five prospective randomized and non-randomized studies support the previously described findings with a significant reduction in 30-day mortality with IVIG (33.7% vs. 15.7%) [22]. The only pediatric study in this meta-analysis was the multicenter, retrospective study on IVIG in children with STSS by Adalat et al. [23]. They reported eight deaths among 29 children with STSS. All children receiving IVIG survived. This underpowered study collected data via questionnaires without validation against patient records or standardized diagnostic criteria and did not adjust for illness severity and other confounders.

Shah et al. performed a larger, more robustly designed pediatric study, which was not included in the above meta-analysis because data were collected retrospectively [16]. This multicenter cohort study included 192 US children (median age 8.2 years) with STSS between 2003 and 2007. Nearly all received clindamycin (94.8%), and 44.6% received IVIG. The overall mortality rate was 4.2% (6% in the IVIG group, 2.8% in the non-IVIG group). A strength is that the study relied on propensity scores to mitigate the risk of indication bias. First, propensity scores were computed to estimate the probability of receiving IVIG given a set of covariates. Then, IVIG recipients and non-recipients were matched on propensity score to create a pseudo-randomized population. After propensity-score matching, the mortality rate was similar in both groups (4.5%). IVIG use did not reduce the length of stay, but was associated with increased total hospital costs, primarily due to the higher drug costs. IVIG costs 45–60€/g, and the typical dose of 2 g/kg translates to roughly 100 €/kg per patient. The authors mentioned the inclusion criteria of discharge diagnoses as a limitation. This study could therefore have included non-STSS patients, less likely to receive IVIG and less likely to die. To minimize misclassification bias, the discharge codes were combined with billing data for intravenous penicillin.

To summarize, the clinical benefit of IVIG in adults and mixed-age populations did not reach statistical significance. Two pediatric studies have been conducted: one with methodological flaws, and one showing no clinical benefit, only increasing costs.

### Clindamycin and IVIG: evidence from real-world practice

There is substantial variability in clindamycin and IVIG use in practice, from which valuable real-world evidence can be derived. In a European multicenter cohort of 320 children, clindamycin was used in 29% (highest in Spain and the UK, with as little as 7% in Switzerland) and IVIG in 4.6% [24]. In contrast, in an Australian surveillance study of 181 children, clindamycin use was much higher: 56% overall, rising to 80% in severe disease and 88.5% in STSS [25]. IVIG was also used more often: 8.8% overall, 20% in severe disease (including STSS), and 38.5% in STSS. Despite variability in management across settings, outcomes were similar, with mortality rates of 2% in the European study and 2.8% in the Australian study.

## Discussion

While β-lactams are the cornerstone of therapy for pediatric iGAS disease, clindamycin and IVIG have been suggested as adjuncts, clindamycin mainly for its potential to inhibit toxin production, and IVIG to counteract the cytokine storm occurring in STSS.

Evidence supporting the benefit of clindamycin and IVIG in pediatric iGAS has been limited by small sample sizes, no adjustment for disease severity, and concerns about generalizability. Variations in the choice of β-lactam and the addition of vancomycin further complicate interpretation. To overcome these limitations, well-conducted multinational and multicenter pediatric studies are needed. While an RCT would be ideal, the rarity of iGAS and STSS, along with ethical concerns about withholding potentially beneficial treatments in critically ill children, presents substantial barriers. Well-conducted observational studies which make use of the increasing amount of electronic health records data can provide valuable complementary evidence. This might be the most realistic way forward, especially when RCTs might not be feasible. An example is the ongoing European PEGASUS study, which has enrolled over 2000 children with iGAS to date.

Based on the available data, the need for adjuncts in pediatric patients with iGAS may seem less urgent compared with adults. This is because mortality ranges from 2 to 8% in children with iGAS disease in general and 5 to 10% in those with STSS, versus 30 to 80% in adults. However, many children require pediatric intensive care unit admission, with rates varying between 22 and 50% [1]. This highlights the importance of considering outcomes beyond mortality in upcoming studies, such as the need for intensive care, which may justify the use of additional therapies in pediatric populations despite generally favorable survival rates.

## Conclusion

Drawing firm conclusions on the use of clindamycin and IVIG as adjuncts in iGAS in children remains challenging due to limited high-quality evidence. Arguments such as “Are you not going to give everything possible if the child is critically ill?” or “Most experts use these therapies” are understandable, pending further robust evidence. This underscores the importance of larger-scale pediatric studies to guide clinical practice.

## Data Availability

No datasets were generated or analysed during the current study.

## Abbreviations

GAS: Group A *Streptococcus*
iGAS: Invasive group A streptococcal disease
IVIG: Intravenous immunoglobulins
NF: Necrotizing fasciitis
RCT: Randomized controlled trial
STSS: Streptococcal toxic shock syndrome
